# The predisposing factors of AKI for prophylactic strategies in burn care

**DOI:** 10.7717/peerj.9984

**Published:** 2020-09-29

**Authors:** Shin-Yi Tsai, Chon-Fu Lio, Shou-Chuan Shih, Cheng-Jui Lin, Yu-Tien Chen, Chia-Meng Yu, Fang-ju Sun, Chien-Feng Kuo, Xiaofeng Jia

**Affiliations:** 1 Department of Laboratory Medicine, Mackay Memorial Hospital, Taipei, Taiwan; 2 Department of Medicine, Mackay Medical College, New Taipei City, Taiwan; 3 Graduate Institute of Biomedical Sciences; Graduate Institute of Long- Term Care, Mackay Medical College, New Taipei City, Taiwan; 4 Department of Health Policy and Management, Bloomberg School of Public Health, The Johns Hopkins University, Baltimore, MD, United States of America; 5 Department of Internal Medicine, Mackay Memorial Hospital, Taipei, Taiwan; 6 Department of Medical Research, Mackay Memorial Hospital, Taipei, Taiwan; 7 Department of Nephrology, Mackay Memorial Hospital, Taipei, Taiwan; 8 Department of Surgery, Mackay Memorial Hospital, Taipei, Taiwan; 9 Department of Plastic Surgery, Mackay Memorial Hospital, Taipei, Taiwan; 10 Institute of Infectious Disease, Mackay Memorial Hospital, Taipei, Taiwan; 11 Biomedical Engineering, Anesthesiology & Critical Care Medicine, The Johns Hopkins University, Baltimore, MD, United States of America; 12 Department of Neurosurgery, Orthopaedics, Anatomy & Neurobiology, University of Maryland at Baltimore, Baltimore, MD, United States of America

**Keywords:** Kidney Disease: Improving Global Outcomes (KDIGO) criteria, Burn injury, Acute kidney injury, Abbreviated Burn Severity Index score, ABSI score, America Burn Association (ABA) sepsis criteria

## Abstract

**Background:**

Acute kidney injury (AKI) is one of the most severe complications of burn injury. AKI with severe burn injury causes high mortality. This study aims to investigate the incidence of and predisposing factors for AKI in burn patients.

**Methods:**

This is a single-center, retrospective, descriptive criterion standard study conducted from June 27, 2015, to March 8, 2016. We used Kidney Disease Improving Global Outcomes criteria to define and select patients with AKI. The study was conducted by recruiting in hospital patients who suffered from the flammable cornstarch-based powder explosion and were treated under primary care procedures. A total of 49 patients who suffered from flammable dust explosion-related burn injury were enrolled and admitted on June 27, 2015. The patients with more than 20% total body surface area of burn were transferred to the intensive care unit. Patients received fluid resuscitation in the first 24 hours based on the Parkland formula. The primary measurements were the incidence of and predisposing factors for AKI in these patients. Demographic characteristics, laboratory data, and inpatient outcomes were also evaluated. The incidence of AKI in this cohort was 61.2% (*n* = 30). The mortality rate was 2.0% (*n* = 1) during a 59-day follow-up period. The multivariate analysis revealed inhalation injury (adjusted OR = 22.0; 95% CI [1.4–358.2]) and meeting ≥3 American Burn Association (ABA) sepsis criteria (adjusted OR = 13.7; 95% CI [1.7–110.5]) as independent risk factors for early advanced AKI.

**Conclusions:**

The incidence rate of AKI was higher in this cohort than in previous studies, possibly due to the flammable dust explosion-related burn injury. However, the mortality was lower than that expected. In clinical practice, indicators of inflammation, including ABA sepsis criteria may help in predicting the risk of AKI in patients with burn injury.

## Introduction

Acute kidney injury (AKI) is one of the most severe complications of burn injury. AKI after severe burn injury causes high mortality as well as increases hospital length of stay (LOS) and cost (Hoste et al., 2006; Steinvall, Bak & Sjoberg, 2008). AKI occurs in one-quarter of patients with severe burn injury (Brusselaers et al., 2010), and mortality ranges from 50% to 100% (Chung et al., 2009; Coca et al., 2007; Mustonen & Vuola, 2008). A trend of improved outcomes in patients with AKI and burn has been reported (Holm et al., 1999). However, few studies have investigated AKI after inflammable dust explosion-related burn injury.

Although the precise etiology of AKI among patients with burn injury remains unclear, it may be multifactorial. One mechanism is that the fulminant inflammatory response after thermal injury causes multiple organ failure, including kidney function impairment (Mehta et al., 2004). In addition, a decrease in the effective intravascular volume during burn shock may also contribute to subsequent AKI (Colpaert & Hoste, 2008). Studies have reported that high total body surface area (TBSA) of burn (Kim et al., 2003); the presence of inhalation injury, (Holm et al., 1999) sepsis, multiple organ failure, use of nephrotoxic drugs, and surgery are risk factors for AKI in patients with burn injury (Palmieri, Lavrentieva & Greenhalgh, 2010). The increased risk of chronic kidney diseases (CKD) after an initial AKI episode may affect patients’ quality of life (Hobson et al., 2009; van Kuijk et al., 2010). Therefore, early identification and intervention are crucial to prevent unfavorable outcomes.

This study investigated predisposing factors for the occurrence of AKI in patients with flammable cornstarch-based powder explosion-related burn injury.

## Materials & Methods

### Study design and patient selection

**Figure 1:**
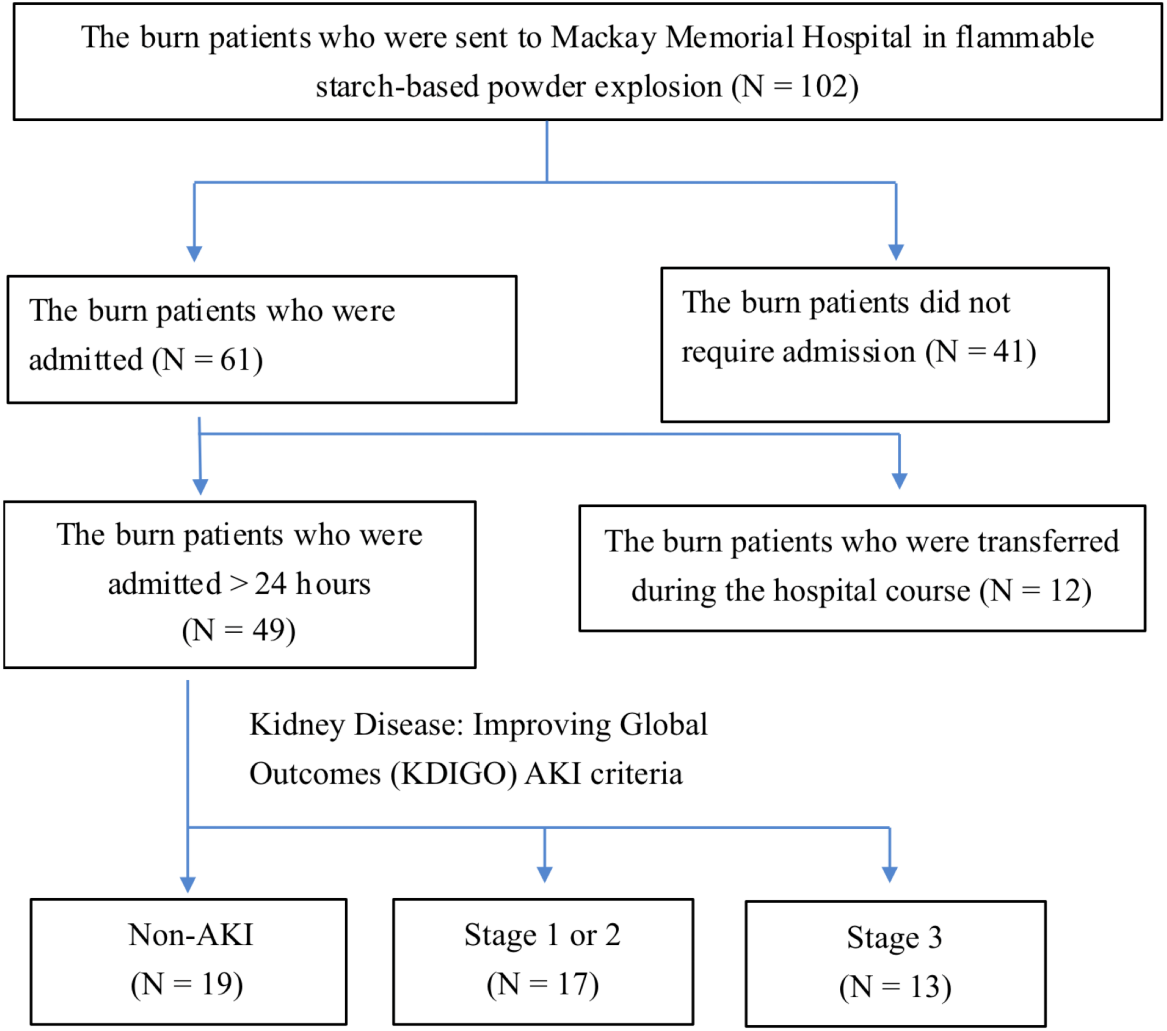
The selection process of the participants in the study.

This retrospective study was conducted at Mackay Memorial Hospital for an 8-month period from June 27, 2015, to March 8, 2016. All consecutive patients were victims of the flammable starch-based powder explosion that occurred in Taiwan on June 27, 2015. The patients who were admitted to Mackay Memorial Hospital were enrolled. We excluded patients who did not require admission, were transferred during the hospital course, and lacked essential lab data to evaluate the kidney function status, and history of comorbid chronic disease (Fig. 1). The standard operating procedure for thermal injury management was followed in the emergency room protocol. For instance, fluid resuscitation in the first 24 hours was based on the following Parkland formula. Patients with more than 20% TBSA of burn were transferred to the intensive care unit (ICU). ICU physicians used central venous pressure (CVP) as a parameter to assess patient’s hydration status, and CVP within the range of 8–12 mmHg was achieved by adjusting the fluid administration according to patients’ clinical status. The ethics committee of Mackay Memorial Hospital granted Ethical approval to carry out the study within its facilities. Written informed consent for study participation and consents for publication were obtained, including any relevant details; and all investigations were performed in accordance with relevant guidelines and regulations, and written informed consent was obtained from all participants and/or their legal guardians.

### Definition of AKI and early-onset AKI

We stratified patients according to the Kidney Disease: Improving Global Outcomes (KDIGO) AKI criteria. A higher stage indicated more severe kidney injury. “Stage 1” was defined as a urine output of <0.5 ml/kg/h for 6–12 h or serum creatinine meeting 1.5–1.9 times baseline or ≥0.3 mg/dl increase. “Stage 2” was defined as a 2.0 to 2.9 times baseline serum creatinine or a urine output <0.5 ml/kg/h for ≥12 h. “Stage 3” was defined 3-fold increase in the baseline serum creatinine level or ≥4.0 mg/dl, or the initiation of renal replacement therapy, or urine output <0.3 ml/kg/h for 24 or more hours, or anuria for ≥12 h. The patients who met any KDIGO classification within 5 days of admission were enrolled in the “early AKI” group and those who did not meet the criteria were enrolled in the “non-AKI” group (Kym et al., 2015; Mustonen & Vuola, 2008). We reviewed records of laboratory data and daily urine output to identify patients with AKI. We used the plasma creatinine level prior to the accident if available as the baseline and to compare it with the initial admission data. When data prior to the accident were not available, the lowest creatinine value at the time of hospitalization was used as the baseline (Kym et al., 2015; Palmieri, Lavrentieva & Greenhalgh, 2010).

### Collection and definition of variables

The patients’ demographic and laboratory data were obtained by reviewing electronic medical records from the hospital information system. The TBSA of the burn was estimated using the Wallace “rule of nines” method. A full-thickness injury was defined as an injury extended to all layers of the skin. Endotracheal tube placement was performed during resuscitation in the patients having evidence of inhalation injury or respiratory failure, and these patients were classified as having inhalation injury. The severity of burn injury was measured using the Abbreviated Burn Severity Index (ABSI) and Acute Physiology and Chronic Health Evaluation II score (Moreno et al., 1999). Nephrotoxicity is a commonly observed adverse effect following the administration of some antibiotics. The administration of aminoglycosides, vancomycin, and colistin preceding the maximum KDIGO class was recorded for each patient (Evans, Feola & Rapp, 1999; Palmieri, Lavrentieva & Greenhalgh, 2010). In our setting, the outcome variables included length of hospital stay (LOS), length of ICU stay, length of mechanical ventilation, infection complication, transfusion, and mortality. Wound infection was considered in patients with clinical symptoms and positive wound culture. Patients with clinical suspicion of pneumonia and having a positive culture from sputum or bronchoalveolar lavage specimen were regarded as respiratory tract infections. Bacteriaemia was defined by positive blood culture in patients with warning signs of sepsis. The laboratory data and vital signs within 48 hours of admission were obtained for analyses.

### American Burn Association (ABA) sepsis criteria in burn patients

The American Burn Association (ABA) developed a scoring system and standardized sepsis criteria for patients with a burn. (Greenhalgh et al., 2007) These criteria are temperature (>39 °C or <36 °C), progressive tachycardia (>110 beats/min), progressive tachypnea (>25 breaths/min without ventilation or >12 breaths/min under ventilation), thrombocytopenia (<100,000/ µL; not until 3d after initial resuscitation), hyperglycemia (untreated plasma glucose >200 mg/dL, an intravenous infusion of >7 units of insulin/h, or >25% increase in insulin requirement over 24 h), and feed intolerance >24 h (abdominal distension, containing residual that is 2 times the feeding rate, or diarrhea >2,500 mL/d). Since thrombocytopenia in the first 2 days is an indicator of hemodilution but not sepsis, we excluded the calculation of thrombocytopenia in this study as the ABA guideline suggested. The patients with burn injury who met ≥3 of these criteria plus documented infection (such as culture-positive infection) were considered for the presence of sepsis. The score obtained was regarded as a parameter for infection in patients with burn injury.

### Statistical analysis

Continuous data were presented as mean ± standard deviation, and dichotomous variables were presented as percentages. An unpaired Student *t*-test and chi-squared test were used to compare demographic and clinical characteristics between the early AKI and non-AKI groups, as well as the “Stage 1 and 2” and “Stage 3” groups (Table 1). The univariate and multivariate analysis were used to determine the risk factors for the onset of advanced AKI (KDIGO stage 2 and 3) within 5 days of admission (Table 2). The above methods were also used to compare patients who met <3 versus ≥3 ABA sepsis criteria on admission (Table 3).

A Mann–Whitney U test was performed when continuous data did not follow a normal distribution. Univariate and multivariate analyses were performed using the binary logistic regression model to identify risk factors for AKI. A Spearman rank test was applied to examine the strength of the association between 2-ranked variables. The associations between AKI severity and the LOS outcome were assessed using Cox proportional hazards regression models with fixed and time-varying covariates (Table 4). In all the comparisons, a *P* value of <0.05 was considered statistically significant. Since the accident is very critical and the society and the people have drawn intensified concerns in Taiwan, all medical teams paid close attention and had the complete medical records of the cases that we recruited, thus, there were few missing data.

**Table 1:** Demographic and clinical characteristics of patients with dust explosion-related burn injury stratified by the severity of early onset of acute kidney injury.

		Early AKI-KDIGO (*N* = 30)			
	**Non-AKI** (*N* = 19)	Stage 1 or 2 (*N* = 17)	**Stage 3** (*N* = 13)	*P***value**^f^	*P* value^g^
**Demographic and clinical characteristicsat admission**					
Age (years; mean ± SD)	23.42(±4.80)	21.82(±2.70)	21.54(±3.90)	0.14	0.97
Gender (Male; N)	11	5	5	0.09	0.87
TBSA (%)^a^	31.53(±20.99)	52.52(±21.56)	65.70( ±16.13)	<0.01	0.15
Present of full thickness burn (%)	84.21%	100%	100%	–	-
Present of inhalation injury (%)	0.00%	41.18%	61.54%	<0.01	0.53
ABSI score^b^ at admission (mean)	6.37(±2.41)	9.30(±2.76)	10.46(±1.85)	<0.01	0.36
Required ICU admission (%)	26.32%	64.71%	69.23%	<0.01	0.97
Initial APACHE II score^c^ (mean)	9.60(±5.73)	14.55(±4.87)	16.10(±4.31)	0.03	0.72
Length of ICU stay	14.20(10.40)	29.72(±15.65)	40.00(±8.72)	<0.01	0.18
Meets of ABA sepsis criteria^d^ at admission (median, IQR)	1.00(1.00)	2.00(2.00)	3.23(±1.17)	<0.01	0.06
Meets ≥ 3 criteria (%)	15.79%	35.29%	76.92%	<0.01	0.17
**Risk factors for AKI development**					
Received operation before maximal KDIGO stage (%)	21.05%	17.65%	7.96%	0.49	0.12
Received nephrotoxic agents before maximal KDIGO stage (%)	26.32%	11.76%	7.69%	0.13	0.77
Insufficient hydration^e^ on day 1(%)	47.37%	76.47%	76.92%	0.46	0.53
Body weight (Kg; median, IQR)	60.00(24.00)	57.10(15.95)	56.90(11.85)	0.63	0.66
**Outcome variables**					
Operation counts during hospitalization (median, IQR)	2.00(4.00)	6.00(4.00)	7.00(3.00)	<0.01	0.09
Wound infection (%)	57.89%	76.47%	100%	0.02	0.12
Respiratory tract infection (%)	5.26%	17.65%	46.15%	0.04	0.29
Bacteriaemia (%)	0.00%	23.53%	61.54%	<0.01	0.12
Whole blood (packs; median, IQR)	0.00(6.00)	0.00(8.00)	2.00(7.00)	0.85	0.68
Packed Red Blood Cell (packs; median, IQR)	24.00(34.00)	14.00(32.00)	10.00(18.00)	0.36	0.63
Frozen Plasma (packs; median, IQR)	18.00(48.00)	16.00(42.00)	10.00(45.00)	0.90	0.88
Albumin supplement (packs; median, IQR)	0.00(16.75)	30.00(61.00)	50.00(36.50)	<0.01	0.38
Length of hospital stay (median, IQR)	25.00(20.50)	61.00(76.50)	91.00(46.00)	<0.01	0.32
Mortality (%)	0%	0%	7.69%	–	–
Recovery time of AKI (days, median, IQR)	–	5.00(6.00)	15.00(17.50)	–	0.01

**Notes.**

SD standard deviation IQR interquartile range

a Total Burn Surface Area of burn.

b Abbreviated Burn Severity Index.

c Acute Physiology and Chronic Health Evaluation II.

d American Burn Association sepsis criteria.

e Volume of sufficient hydration is estimated by Parkland formula.

f Comparison between “Non-AKI” and “Early AKI” group.

g Comparison between ‘KDIGO Stage 1 or 2’ and “Stage 3’ group.

Statistical analyses were performed using the SPSS software, Version 17.0 (SPSS Inc., Chicago, IL, USA).

## Results

In total, 49 patients with dust explosion injury were enrolled. The average age of consecutive patients was 22.4 years. The mean follow-up period was 59 days. The average TBSA percentage of the burn was 47.9% (±24.2%). The mean ABSI score was 8.5 (±0.42). Most of the patients (93.9%) had full-thickness burn injury, and 18.4% had inhalation injury. However, none of them had other comorbidities before the accident. The incidence of AKI in our cohort was 61.2% (*n* = 30). 12.2% (*n* = 6), 22.4% (*n* = 11) and 26.5% (*n* = 13) of patients had the KDIGO-AKI classification stage 1, 2 and 3 respectively. The mortality rate was 2.04% (1/49) in the overall cohort and 6.7% in the KDIGO-AKI stage 3 cohort. Only one male patient with 55% TBSA of burn died due to multiple organ failure and shock 11 days after admission. In addition, he was the only patient with kidney failure requiring hemodialysis therapy (1/13; 6.7%).

**Table 2:** Univariate and multivariate analysis to determine the risk factors for onset of advanced AKI (KDIGO stage 2 and 3) within 5 days of admission.

	Univariate analysis^a^			Multivariate analysis^b^		
	Unadjusted odd ratio	95% confidence interval	*P* value	Adjusted odd ratio	95% Confidence interval	*P* value
**Age**	0.92	0.79–1.07	0.25	0.81	0.58–1.12	0.20
**Gender**						
Female	1	(Reference)		1	(Reference)	
Male	0.65	0.21–2.03	0.46	1.70	0.24–11.87	0.59
**TBSA% of burn**						
Non-severe; <50%	1	(Reference)		1	(Reference)	
Severe; ≥50%	18.00	4.05–80.02	<0.01	2.60	0.30–22.35	0.38
**Inhalation injury**						
Absent	1	(Reference)		1	(Reference)	
Present	33.60	3.88–290.98	<0.01	22.00	1.35–358.21	0.03
**ABSI score**	1.65	1.24–2.20	<0.01	–	–	–
**ABA sepsis criteria**^c^						
Meets < 3 criteria	1	(Reference)		1	(Reference)	
Meets ≥ 3 criteria	9.21	2.18–38.86	<0.01	13.66	1.69–110.46	0.01
**Operation before maximal KDIGO stage**						
No	1	(Reference)		–	–	–
Yes	0.29	0.05–1.60	0.15	–	–	–
**Nephrotoxic agents before maximal KDIGO stage**						
No	1	(Reference)		–	–	–
Yes	0.22	0.04–1.20	0.11	–	–	–
**Sufficient hydration on day 1**^d^						
No	1	(Reference)		–	–	–
Yes	0.52	0.10–2.77	0.44	–	–	–

**Notes.**

a Binary univariate logistic regression analysis.

b Variables in multivariate analysis, including age, gender, TBSA% of burn, inhalation injury and meeting ≥ 3 of ABA sepsis criteria. ABSI score was eliminated in multivariate analysis due to multicollinearity between ABSI score, TBSA% and inhalation injury status.

c American Burn Association sepsis criteria.

d Volume of sufficient hydration is estimated by Parkland formula.

**Table 3:** Laboratory characteristics of patients with dust explosion-related burn injury stratified by the number of ABA sepsis criteria met on admission.

	**Meets** < 3 criteria (*N* = 30)	Meets ≥ 3 criteria (*N* = 19)	*P* value
White blood cell count (1 × 10^3^ cells/µL; mean ± SD)	18.37 (±11.54)	29.65 (±13.04)	<0.01
Blood Urea Nitrogen (mg/dL; mean ± SD)	10.78 (±4.25)	13.47 (±3.79)	0.03
Creatinine (mg/dL; mean ± SD)	0.84 (±0.23)	1.07 (±0.36)	0.01
Estimated GFR (ml/min/1.73 m^2^; mean ± SD)	102.35 (±37.12)	84.87 (±38.07)	0.16
Potassium (mmol/L; median, IQR)	3.90 (0.40)	4.30 (0.60)	0.01
Albumin (g/dL; median, IQR)	1.70 (1.50)	1.50 (0.50)	0.04
Fasting blood glucose (mg/dL; mean ± SD)	128.27 (±33/09)	155.37 (±44.76)	0.02
Mean arterial pressure (mmHg; mean ± SD)	73.96 (±12.28)	65.04 (±14.26)	0.02

**Notes.**

SD standard deviation IQR interquartile range

**Table 4:** Associations between length of hospital stay and occurrence of early acute kidney injury adjusted by initial burn injury severity.

	Adjusted Hazard ratio		**95% Confidence interval**	
	Time to hospital discharge alive	*P* value	Lower	Upper
Initial burn injury severity				
ABSI^a^	0.67	<0.01	0.56	0.80
Occurrence of acute kidney injury (AKI) within 5 days of admission.				
No AKI	1.00	–	–	–
KDIGO^b^ stage 1 or 2	0.41	0.04	0.17	0.96
KDIGO stage 3	0.27	<0.01	0.11	0.69

**Notes.**

a Abbreviated Burn Severity Index.

b Kidney Disease: Improving Global Outcomes (KDIGO) criteria for acute kidney injury.

The results of the comparison of demographic and clinical characteristics between different groups are listed in Table 1. Compared with the patients without AKI, those with early AKI tended to have a higher TBSA percentage of burn, ABSI score, and number of surgeries; more patients meet ≥3 ABA sepsis criteria on admission; longer hospital and ICU LOS; and higher prevalence of inhalation injury (*P* < 0.05). Patients with early AKI had a notably greater prevalence of subsequent infections at different sites, including skin wounds (*P* = 0.02), respiratory tract (*P* = 0.04), and blood (*P* = 0.01). Although the volume of various blood transfusions was comparable between groups, the patient with kidney injury received an infusion with higher albumin content (*P* < 0.01). Among all the variables in Table 1, only the recovery time of AKI significantly differed among different AKI severity groups. The mean recovery time of AKI in the “injury” and “failure” groups was 5.7 and 16.0 days, respectively. The log-rank test revealed that the recovery time significantly differed among different AKI severity groups (*P* = 0.01).

As listed in Table 2, the univariate analysis revealed that a total body surface area of burns ≥ 50% (OR = 18.0; 95% CI [4.1–20.0]), inhalation injury (OR = 33.6; 95% CI [3.9–291.0]), Abbreviated Burn Severity Index score (OR = 1.7; 95% CI [1.2–2.2]), and meeting ≥3 American Burn Association (ABA) sepsis criteria (OR = 9.2; 95% CI [2.2–38.9]) were associated with a risk of early advanced AKI. The multivariate analysis revealed inhalation injury (adjusted OR = 22.0; 95% CI [1.4–358.2]) and meeting ≥3 ABA sepsis criteria (adjusted OR = 13.7; 95% CI [1.7–110.5]) as independent risk factors for early advanced AKI.

Since ABA sepsis criteria is a clinical parameter reflecting the severity of inflammation, the patients who meet a higher count of ABA criteria may present different laboratory characteristics. Indeed, we found patients with meets ≥ 3 criteria had higher white blood cell count, lower blood pressure, and worse baseline renal function during initial admission assessment, as summarized in Table 3.

**Figure 2:**
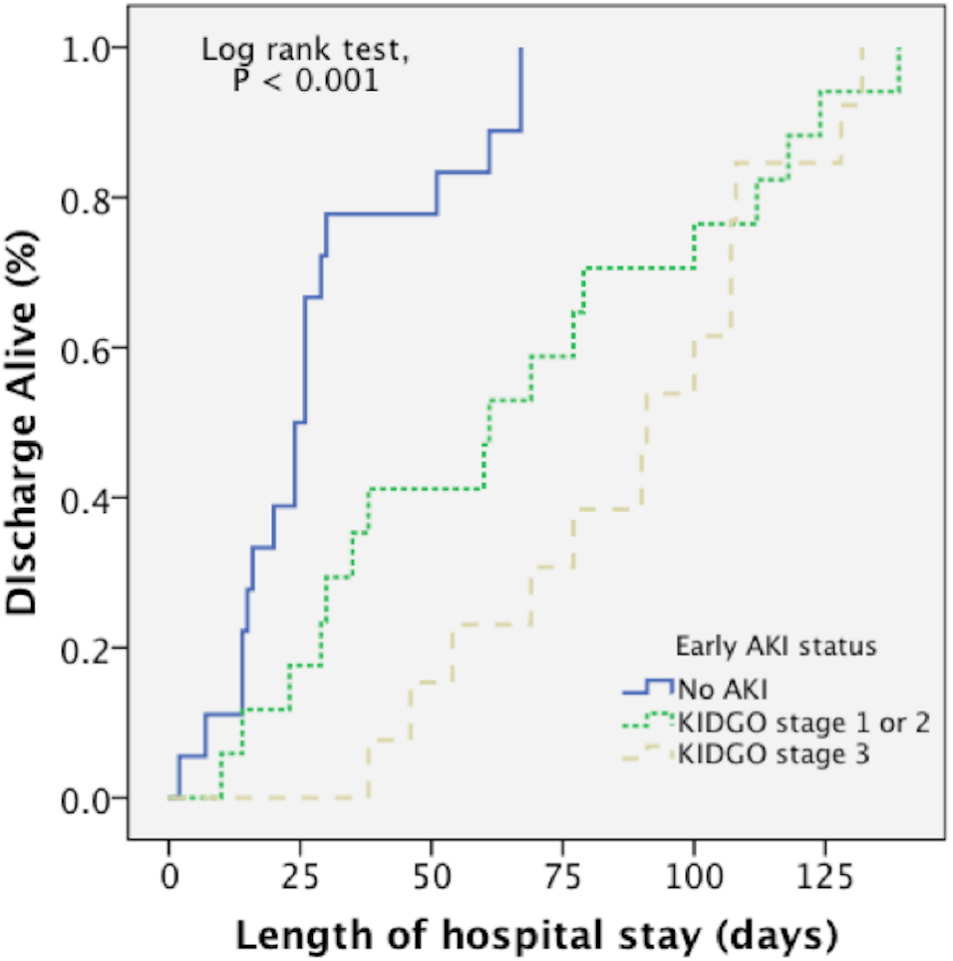
A Kaplan–Meier curve demonstrated that there was a longer length of hospital stay in patients with early AKI. (Log rank test, *P* < .001).

A Kaplan–Meier curve (Fig. 2) demonstrated that there was a longer length of hospital stay in patients with early AKI (Log-rank test, *P* < 0.001). To clarify whether the extent of the injury or the AKI *per se* affects the outcomes, we included ABSI as confounding factors to investigate the impact of AKI on the length of hospital stay by a multivariate Cox regression model (Table 4). It was clear that a higher ABSI score (adj. HR, 0.67; 95% CI [0.56–0.80]; *P* < 0.001) was associated with significantly lower hazards of hospital discharge, suggesting a longer time to hospital discharge. Nonetheless, the development of early AKI had a significant impact on the length of hospitalization. Moreover, difference in AKI severity, such as “stage 1 or 2” (adj. HR, 0.41; 95% CI [0.17–0.96]; *P* = 0.04) and “stage 3” (adj. HR, 0.70; *P* = 0.006), were also correlated with outcomes independently.

## Discussion

To the best of our knowledge, this is the largest study describing the characteristics of flammable cornstarch-based powder explosion-related burn injury and examining risk factors for AKI in these patients using KDIGO-AKI criteria. Additionally, this is the first study to apply sepsis criteria specifically for a burn injury in AKI research. Previous studies have reported TBSA of burn as an independent risk factor for AKI. However, our study firstly demonstrated that the effect of inflammation was greater than that of TBSA percentage of burn on AKI. Thus, AKI development may be dependent on inflammation rather than on TBSA.

The onset of AKI has been published to be associated with the baseline severity of the burn (Holm et al., 1999; Kim et al., 2003; Palmieri, Lavrentieva & Greenhalgh, 2010). Our results showed inhalation injury and ABSI scores were significant predictors of early AKI, concurring the results in other settings. The previous study showed the use of nephrotoxic drugs and surgical operations were risk factors of AKI in burn-injured (Palmieri, Lavrentieva & Greenhalgh, 2010). Cho et al. (2012) found that subsequent elevation of serum creatinine after colistin administration was within the normal range in major burn patients. Hence, mild renal impairment after administration of nephrotoxic agents may not adequately meet the threshold of being classified as AKI.

To the best of our knowledge, this is the first study reporting ABA sepsis criteria as a risk factor for early AKI. Meeting a high number of ABA sepsis criteria reflects more fulminant inflammation. This result is compatible with that of a previous study that reported a strong correlation between the extent of sepsis and the incidence of AKI in critically ill patients (Riedemann, Guo & Ward, 2003). Pathogenesis may involve the release of TNF, endothelin, catecholamines, vasopressin, and angiotensin-II in those with severe sepsis. This cytokine storm can trigger renal vasoconstriction leading to inadequate tissue perfusion (Schrier & Wang, 2004). In addition, microthrombi caused by the activation of the cytokine storm and procoagulation state can lead to kidney ischemia (Reinhart et al., 2002). Our results are consistent with a previous study that reported inhalation injury as an independent risk factor for AKI (Holm et al., 1999). Inhalation injury can upregulate several immune mediators such as interleukin (IL)-1RA, IL-6, IL-8, granulocyte colony-stimulating factor, and monocyte chemotactic protein 1 (Davis et al., 2013). Moreover, we have investigated the impact of the thermal injury on the risk of chronic fatigue syndrome (Tsai et al., 2018) through the numerous proinflammatory cytokines leading to the Systemic inflammatory response syndrome (SIRS) as the first phase of thermal injury (Nielson et al., 2017). Systemic inflammation in patients with a large burn area and inhalation injury may play a crucial role in the pathogenesis of AKI.

Because this dust explosion accident was a mass casualty event, with more than 500 people being injured, chaos in rescue and transportation seemed to be inevitable. Therefore, most of the victims stayed at the site to aid others. However, several of these victims had used contaminated water from a water channel in the park and Tan-Shui river to wash their wounds. Thus, cases of patients with invasive *Vibrio* species infection have been reported. Hsieh et al. suggested that contamination of a burn wound by river water is involved in the pathogenesis of the entry of *Vibrio* species (Hsieh et al., 2015). In our study, the AKI incidence among patients with dust explosion-related burn injuries was 61.2%. This incidence rate is higher than that reported in previous studies (Kim et al., 2003; Steinvall, Bak & Sjoberg, 2008). Because wound infections exacerbate the inflammatory process and cytokine storm, the use of contaminated water for cleaning wounds might have been one reason for the high AKI incidence in our study.

Our results revealed that the hospital mortality rate in patients with acute kidney failure was 7.7%. The low mortality in our study may have been attributable to the young, healthy study population. Because economic status was previously shown to be negatively correlated with burn mortality, (Peck & Pressman, 2013) the crisis management of local policymakers might be one of the reasons for high survival. All the medical expenses of the patients were covered by the National Health Insurance Program. In addition, early interventions such as prompt fluid resuscitation, eschar excision, and wound closure can improve mortality in patients with burn injury (Klein et al., 2007). The time required to transfer children and patients with inhalation injury to a burn centre negatively affects their outcomes (Cassidy et al., 2015; Sheridan et al., 1999). Cheng et al. (2016) reported that the effective mass casualty management system initiated immediately after the accident by the Ministry of Health and Welfare of Taiwan played a vital role in triaging and transferring patients to suitable hospitals across Taiwan in a short period of time, eventually helping to minimize the casualties.

The precise mechanism of AKI occurrence in patients with burn injury is not fully understood. Hypovolemia, cytokine release, denatured proteins, and nephrotoxic drugs might be involved in the physiopathology of AKI (Ibrahim et al., 2013). In the early stage of thermal injury, damage to the skin barrier reduces the effective intravascular volume, causing renal damage (Colpaert & Hoste, 2008). A study reported that TBSA of burn is a predisposing factor for AKI (Kim et al., 2003). Since fluid loss occurs as a result of evaporation and exudation from burn wounds, hypovolemia may trigger or exacerbate the kidney damage in this scenario. In our study, relatively low blood pressure and fast heartbeat were found in the patients with higher severity of burn injury at admission, suggesting that a relatively depleted circulatory volume may be a common phenomenon in patients with AKI.

Our study had several strengths. We considered the homogeneity of the entire cohort and selected only victims of the Formosa Fun Coast explosion in–hospital treatment. All the patients included were young and had no history of chronic diseases. We believe the homogeneity of the enrolled patients can help us better understand the influence of dust explosion on the outcomes (Tsai et al., 2020). Furthermore, based on previous studies of patients with burn and AKI, we examined possible factors that may interfere with AKI incidence. For instance, operation, volume status, and use of nephrotoxic agents were considered in the analyses.

Our study had some limitations. First, the number of enrolled patients was relatively small; thus, drawing definite conclusions was relatively difficult. Second, laboratory data were not obtained at the same time because of the retrospective nature of this study. We might have missed the peak value of some data, which may have affected the assessment of the recovery time of AKI. Nevertheless, the deviation was only a maximum of 1–2 days. Third, AKI detection is crucial in patients with thermal injury. However, our routine measurements of creatinine level and urine output might not be sensitive to the rapid fluctuation of kidney function in the acute phase (McCullough et al., 2013). Several biomarkers such as neutrophil gelatinase-associated lipocalin, urinary kidney injury molecule-1, IL-18, cystatin C, and lactate dehydrogenase have been proposed for AKI detection (Bonventre et al., 2010; Sen et al., 2015; Zager, Johnson & Becker, 2013). Future studies should utilize these modern biomarkers in order to recognize the onset of AKI precisely.

In conclusion, the incidence rate of early AKI was relatively high in patients with flammable dust explosion-related burn injuries. However, lower-than-expected mortality was observed. We should be aware of AKI development in patients with inhalation injury and those meeting ≥3 ABA sepsis criteria. Our results emphasized the importance of systemic inflammation on AKI development in major burn patients.

##  Supplemental Information

10.7717/peerj.9984/supp-1 Data S1 Raw_data_AKI Each data point indicates the age, sex, the transfusion of different blood components, APACHEII, AKI status, bacteremia and etc across 49 patients

## Additional Information and Declarations

### Data Availability

The following information was supplied regarding data availability: Data are available at figshare: Chen (2020): Data_Burn_AKI.xlsx. figshare. Dataset https://doi.org/10.6084/m9.figshare.12782534.v1.
